# Structure and Spin-Glass Magnetism of the Mn_*x*_Ni_2_Zn_11–*x*_ Pseudobinary γ-Brasses at Low Mn Contents

**DOI:** 10.1021/acs.inorgchem.1c01418

**Published:** 2021-08-02

**Authors:** Sivaprasad Ghanta, Anustoop Das, Partha Pratim Jana, Stanislav Vrtnik, Darja Gačnik, Jože Luzar, Andreja Jelen, Primož Koželj, Magdalena Wencka, Janez Dolinšek

**Affiliations:** † Department of Chemistry, Indian Institute of Technology, 721302 Kharagpur, India; ‡ J. Stefan Institute, Jamova 39, SI-1000 Ljubljana, Slovenia; § Faculty of Mathematics and Physics, University of Ljubljana, Jadranska 19, SI-1000 Ljubljana, Slovenia; ∥ Institute of Molecular Physics, Polish Academy of Sciences, Smoluchowskiego 17, PL-60-179 Poznań, Poland

## Abstract

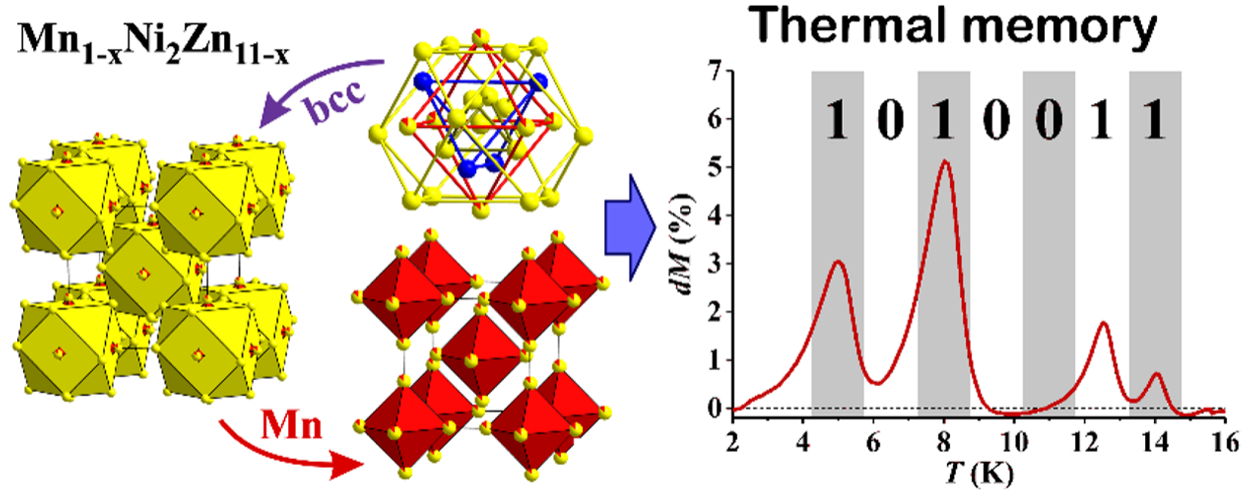

The pseudobinary Mn_*x*_Ni_2_Zn_11–*x*_ γ-brass-type phases at low Mn dopant levels (*x* = 0.1–0.5) were investigated. Crystal structures were determined for the two loading compositions of *x* = 0.3 and 0.5. The structures were solved in the cubic space group of *I*43*m* and are described in close analogy to the Ni_2_Zn_11_ parent γ-brass that is based on the 26-atom cluster, consisting of inner tetrahedron (IT), outer tetrahedron (OT), octahedron (OH), and cuboctahedron (CO). The refined site occupancies of the Mn_*x*_Ni_2_Zn_11–*x*_ (*x* = 0.3, 0.5) reveal that the cluster center, which is empty in the Ni_2_Zn_11_, shows a partial occupation by Zn, with a partial depletion of Zn at the IT sites. The OH sites show a mixed Zn/Mn occupation. The OT and CO sites remain intact with respect to Ni_2_Zn_11_. Magnetic properties were studied for the Mn_0.3_Ni_2_Zn_10.7_ composition. The temperature-dependent zero-field-cooled and field-cooled magnetization, the ac susceptibility, the *M*(*H*) hysteresis curves, the thermoremanent magnetization, and the memory effect demonstrate typical broken-ergodicity phenomena of a magnetically frustrated spin system below the spin freezing temperature *T*_f_ ≈ 16 K. The Mn_0.3_Ni_2_Zn_10.7_ γ-brass phase classifies as a spin glass, originating predominantly from the random distribution of diluted Mn moments on the octahedral partial structure.

## Abstract 2

In a search for new pseudobinary/ternary γ-brass-type phases, we synthesized a series of Mn_*x*_Ni_2_Zn_11−*x*_ compounds at low Mn doping levels (*x* = 0.1−0.5) and determined the structures for two compositions *x* = 0.3 and 0.5. Magnetic measurements show that the Mn_0.3_Ni_2_Zn_11.7_ γ-brass is a spin glass.

## 1 Introduction

Complex metallic alloys (CMAs) are a class of intermetallic compounds that possess giant unit cells comprising from several tens up to many thousands of atoms arranged in well-defined atomic clusters of different polyhedral symmetry.^1,2^ CMA structures possess various types of disorder, including configurational disorder due to a statistically varying orientation of a particular subcluster, chemical (substitutional) disorder, partial site occupation (occupancy smaller than 1), and split occupation, where two neighboring sites are alternatively occupied because they are too close in space. Many CMAs possess a solid-solution range, so that the composition of a single phase can be varied. The category of CMAs includes the family of γ-brasses, containing 52 atoms in the unit cell.^3^ The structure of γ-brass-type phases is described in terms of a 26-atom cluster, made up of four successive shells. Starting from the empty cluster center (CC), the first shell is the inner tetrahedron (IT), followed by outer tetrahedron (OT), octahedron (OH), and cuboctahedron (CO). On the basis of the space group, γ-brasses are divided into four families:^3^ (1) *I*-cell-type γ-brasses with space group *I*43*m* form a body-centered cubic (bcc) lattice with two identical 26-atom clusters in the unit cell, centered at the highly symmetric positions (0,0,0) and (1/2,1/2,1/2), (2) *P*-cell type with space group *P*43*m* adopt a CsCl structure composed of two different 26-atom clusters, (3) *F*-cell type with space group *F*43*m* form superstructures, and (4) *R*-cell type with space group *R*3*m* (a subgroup of *P*43*m*) are rhombohedral. The *I*-cell and *P*-cell types are the most numerous ones, found in 24 binary alloy systems.^3^

Binary γ-brasses were widely investigated in the past, both experimentally and theoretically, and the state of the art in the field is summarized in the book by Mizutani.^3^ Within the finite solid solution range of the γ-brass phase, the stoichiometric compositions can adopt one of the A_2_B_11_, A_4_B_9_, A_5_B_8_, A_7_B_6_, and A_10_B_3_ formulas with space group *I*43*m* and a combination of these two with space group *P*43*m*. According to the classification in terms of a combination of constituent elements in the periodic table, the *I*- and *P*-cell γ-brasses are divided into three groups. Group I comprises 11 combinations of a monovalent noble metal (Cu, Ag) and a polyvalent element, whose valency is well-defined. Group II also includes 11 γ-brasses, which consist of 3d transition-metal elements like TM = V, Mn, Fe, Co, and Ni and either divalent elements Be, Zn, Cd or trivalent elements Al, In. Group III includes γ-brasses consisting of a combination of two nontransition metal elements like Ag, Li, and Pb.

The γ-brass phases belong to the class of Hume–Rothery phases, where the crystal structure achieves stability at a specific valence electron concentration of *e*/*a*.^3−7^ A pseudogap in the electronic density of states (DOS) is formed at the Fermi energy ε_F_, which efficiently lowers the kinetic energy of the conduction-electron system, making the structure more stable. γ-Brasses from the group I are stabilized at the value *e*/*a* = 21/13 = 1.615. For the group II, the situation is less clear, because the *e*/*a* value for the TM is not a-priori known. Theoretical band calculations using the Full-Potential Linearized Augmented Plane Wave (FLAPW) method applied to the stoichiometric TM_2_Zn_11_ (TM = Fe, Co, Ni, Pd) γ-brasses from the group II have yielded *e*/*a* values between 1.70 and 1.80,^6^ which is considered still acceptable to claim that the stability of these alloys follows the Hume–Rothery electron concentration rule. The γ-brass phase field for the TM_2_Zn_11_ family is extended over 15–30 atom % of the TM element.

Inspired by the knowledge and understanding of the stabilization mechanism of binary γ-brasses, the research has been extended to ternary γ-brass phases. New pseudobinary/ternary γ-brass phases were unraveled in the Co–Pd–Zn,^8^ Fe–Pd–Zn,^9^ and Ni–Zn-X (X = In, Ga)^10^ systems, starting from the knowledge of electronic structures of the binary Co_2_Zn_11_, Fe_2_Zn_11_, Pd_2_Zn_11_, and Ni_2_Zn_11_. The Co–Pd–Zn and Fe–Pd–Zn ternary compounds show weak ferromagnetic or ferrimagnetic behavior, unlike their binary parent compounds. Recently, the phase diagram of the ternary Mn–Ni–Zn system has been studied.^11,12^ Three new ternary compounds, denoted as T, τ_1_, and τ_1_, were found to exist at 400 °C, and extended single-phase regions of the phases Mn_5_Zn_21_, Ni_2_Zn_11_, NiZn_3_, and NiZn were observed. In this work, we investigate experimentally the γ-brass-type Mn–Ni–Zn pseudobinary/ternary phases based on the Ni_2_Zn_11_ parent compound.^13^ By introducing Mn as a dopant to the Ni_2_Zn_11_, the atomic radius of Mn (*r*_Mn_ = 127 pm) is closer to Zn (*r*_Zn_ = 134 pm) than Ni (*r*_Ni_ = 124.6 pm). The Pauling electronegativity of Mn (1.55) is also closer to Zn (1.65) than Ni (1.91). For those reasons it can be assumed that Mn will preferentially substitute for Zn. Here we concentrate on the system Mn_*x*_Ni_2_Zn_11–*x*_ at low Mn dopant levels (*x* = 0.1–0.5), by determining the crystallographic structure for two nonstoichiometric compositions and the physical properties, with the emphasis on magnetism.

## 2 Results

### 2.1 Structure Determination and Description

The materials, synthesis, and characterization by powder X-ray diffraction (PXRD), single-crystal X-ray diffraction (SCXRD), and energy-dispersive X-ray spectroscopy (EDS) are detailed in the Experimental Section. The phase purity of the samples was verified by PXRD and Rietveld refinement plots, confirming that the entire series of the Mn_*x*_Ni_2_Zn_11–*x*_ (*x* = 0.1–0.5) compounds were single-phase, belonging to the γ-brass-type phase *I*43*m*.

**Figure 1:**
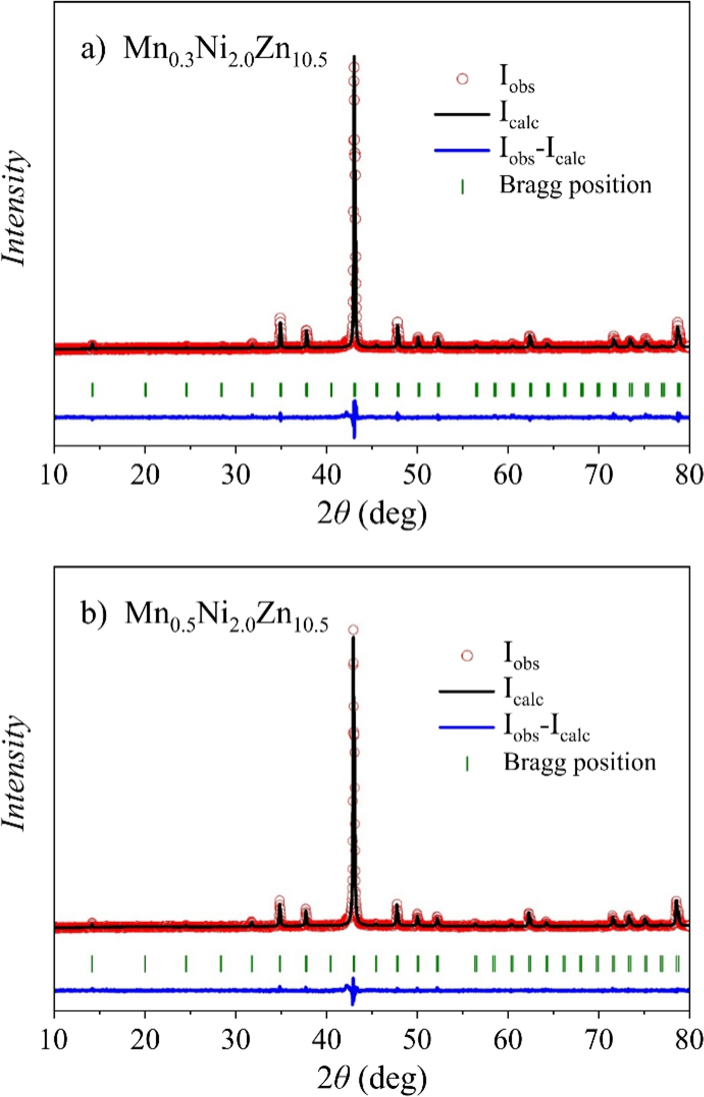
Powder XRD spectra and Rietveld refinement plots for the loading compositions (a) Mn_0.3_Ni_2.0_Zn_10.7_ (GOF = 1.34, *R*_all_ = 5.58, *wR*_all_ = 5.86) and (b) Mn_0.5_Ni_2.0_Zn_10.5_ (GOF = 1.30, *R*_all_ = 2.95, *wR*_all_ = 3.20).

The PXRD spectra and the Rietveld refinement plots for the loading compositions Mn_0.3_Ni_2.0_Zn_10.7_ (C1) and Mn_0.5_Ni_2.0_Zn_10.5_ (C2) are shown in Figure 1 with the goodness of fit (GOF) indicated. The crystal structures were subsequently determined by SCXRD for these two compositions. The complete crystallographic data are presented in Table 1 (additional crystallographic information is available regarding the Accession Codes), whereas the final atomic coordinates, site occupancies, and isotropic displacement parameters are given in Table 2.

**Table 1:** X-ray Crystallographic Data for the Crystals of Loading Compositions Mn_0.3_Ni_2.0_Zn_10.7_ (C1) and Mn_0.5_Ni_2.0_Zn_10.5_ (C2)

	C1	C2
chemical formula	Mn_1.56__(10)_Ni_8_Zn_41.54__(3)_	Mn_1.96__(7)_Ni_8_Zn_41.55__(2)_
chemical formula (atom %)	Mn_3.05_Ni_15.66_Zn_81.29_	Mn_3.81_Ni_15.53_Zn_80.66_
EDS formula (atom %)	Mn_3.3__(1)_Ni_14.81__(1)_Zn_81.9__(1)_	Mn_3.5__(1)_Ni_15.0__(2)_Zn_81.5__(2)_
loading composition (atom %)	Mn_2.31_Ni_15.38_Zn_82.32_	Mn_3.85_Ni_15.38_Zn_80.77_
Pearson symbol	*cI∼*51	*cI∼*52
crystal system	cubic	
space group; *Z*	*I*43*m* (217); 1	
*a*, Å	8.9420(2)	8.9628(2)
*V*, Å^3^	715.00(3)	720.00(3)
ρ_calc_, g cm^–3^	7.597	7.590
μ, mm^–1^	39.811	39.681
crystal color	silvery with metallic luster	
data collection	four-circle diffractometer	
diffractometer	Bruker Photon II	
radiation; wavelength, Å	Mo Kα; 0.71073	
monochromator	graphite	
*T*, K	293	
θ_min_ – θ_max_, deg	3.22–30.39	3.21–30.51
reflns measured	12 933	9178
index range	–12 ≤ *h* ≤ 12 –12 ≤ *k* ≤ 12 –12 ≤ *l* ≤ 12	–12 ≤ *h* ≤ 12 –12 ≤ *k* ≤ 12 –12 ≤ *l* ≤ 12
data reduction/abs correction	multiscan	
crystal size, mm	0.08 × 0.04 × 0.02 0.1 × 0.08 × 0.02	
unique reflns	229	234
*R*_int_	0.0618	0.0628
structure solution/refinement	JANA2006 package program	
structure solution	Superflip	
No. reflns used	229	234
No. variables	22	22
observed reflns (*I* > 3σ(*I*))	226	230
*R*(*F*^2^ > 3σ(*F*^2^))	0.0236	0.0168
*R*(*F*) (all data)	0.0241	0.0173
*K*^a^	0.0004	0.0004
*wR*(*F*^2^) (all data)	0.0735	0.0454
GOF (all)	1.67	1.67
Δ*ρ*_min_/Δ*ρ*_max_ (e Å^–3^)	–0.59/2.25	–1.48/0.56

a Weighting scheme based on measured standard uncertainty *w* = 1/(σ2(*I*) + *kI*2).

**Table 2:** Atomic Coordinates, Site Occupancies, and Equivalent Isotropic Displacement Parameters of Mn_0.3_Ni_2.0_Zn_10.7_ (C1) and Mn_0.5_Ni_2.0_Zn_10.5_(C2)

crystal	atom	Wyckoff	occupancy	*x*	*y*	*z*	*U*_eq_,^a^ Å^2^
C1	Zn0	2a(CC)	0.107(13)	0.5	0.5	0.5	0.016(4)
C2			0.082(11)	0.5	0.5	0.5	0.030(6)
C1	Zn1	8c(IT)	0.861(7)	0.605 28(10)	0.394 72(10)	0.605 28(10)	0.0136(2)
C2			0.913(5)	0.605 20(7)	0.394 80(7)	0.605 20(7)	0.013 62(17)
C1	Ni1	8c(OT)	1	0.671 98(9)	0.328 02(9)	0.328 02(9)	0.009 16(19)
C2			1	0.671 83(6)	0.328 17(6)	0.328 17(6)	0.009 47(13)
C1	Zn2/Mn2	12e(OH)	0.87(3)/0.13	0.852 47(16)	0.5	0.5	0.0166(3)
C2			0.84(2)/0.16	0.853 20(11)	0.5	0.5	0.0144(2)
C1	Zn3	24g(CO)	1	0.954 96(9)	0.306 26(7)	0.306 26(7)	0.0163(2)
C2			1	0.955 83(7)	0.306 69(5)	0.306 69(5)	0.015 81(16)

a *U*_eq_, Å^2^ is defined as one-third of the trace of the orthogonalized *U*^ij^ tensor.

The crystal structures were solved in the cubic space group *I*43*m* (No. 217). The structure solution has generated four crystallographically independent positions in the unit cell. The preliminary stage of refinement for the crystal C1 converged at the *R*(*F*) value of 4.8%. It was challenging to identify the exact atomic positions of Ni and Zn in the crystal structure on the basis of electron density due to their very similar X-ray scattering factors. Thus, at this stage, the positions of Ni and Zn were allocated based on the model established for ordered γ-brass type Ni_2_Zn_11_.^14,15^ Of the four crystallographic sites, one 8c site was assigned to Ni, whereas the remaining three (8c, 12e, 24g) were assigned to Zn, and the subsequent refinement converged at the *R*(*F*) value of 4%. The 12e site discerned a relatively larger isotropic atomic displacement parameter (ADP) of ∼0.021 Å^2^ with respect to the average value. An independent refinement of the 12e site assigned as Zn resulted in a site occupancy factor (SOF) lower than unity, whereas the site assigned as Mn led to the occupancy (SOF) higher than unity. Hence, the 12e site was modeled by partially replacing Mn for Zn (Zn2/Mn2) assuming these sites to be fully occupied overall. The subsequent refinement resulted in the *R*(*F*) value of 3.5%. The 8c site occupied by Zn also discerned a slightly larger isotropic ADP (∼0.018 Å^2^) and was tested for partial occupancy. At this stage, additional residual electron density was identified at the 2a site in the cluster center (CC) located at (0.5, 0.5, 0.5) and included as Zn. The 2a (Zn) site was also examined for partial occupancy, as the distance between the 2a and 8c sites is too short (∼1.63 Å). The local structural disorder was modeled by introducing split positions. In no case does the sum of site occupancy factors (SOFs) of two such sites exceed unity, but the splits are explainable in terms of a set of locally ordered clusters. Both sites were independently refined, following the relation of SOF(Zn(CC)) + SOF(Zn(IT)) ≈ 1.00. The harmonic ADPs for all atoms including the isotropic extinction correction were taken into account at the final stage of the refinement, and the *R*(*F*) value converged at 2.4%. The SCXRD refined model for the C1 crystal was used as the initial model for the refinement of the SCXRD data collected for the C2 crystal. The refined (*Z* = 4) compositions Mn_0.396_Ni_2_Zn_10.38_ for the C1 and Mn_0.491_Ni_2_Zn_10.376_ for the C2 were checked by EDS, and good matching was obtained (Table 1). Each structure (C1 and C2) was tested for possible twinning by an inversion and absolute configuration. The Flack parameters for C1 and C2 after the refinement resulted in −0.1(2) and −0.03(16), respectively. Hence, the structures are not twinned by an inversion. It is worth mentioning that these data were tentatively truncated to test for any trend in the occupancy of the Zn2/Mn2 site. No such trend was detected, that is, the data are stable toward a high-order truncation. An artificial truncation of the data to lower reciprocal space vectors resulted in similar values for the occupancy of the Zn2/Mn2 site.

**Figure 2:**
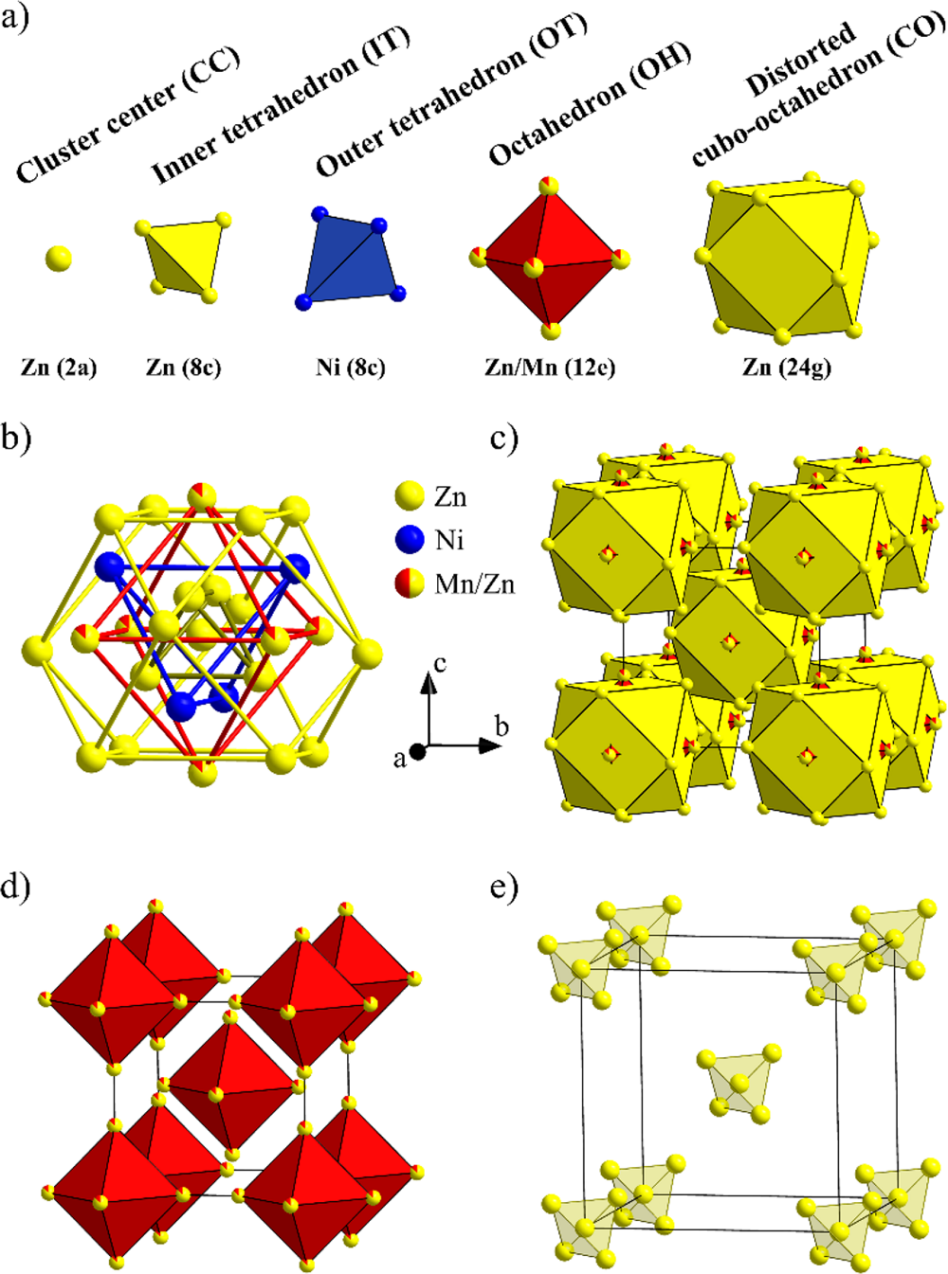
Crystal structure for the composition of Mn_0.396_Ni_2_Zn_10.38_ (crystal C1). (a) Successive shells of the distorted γ-cluster, with the OH sites showing mixed Zn/Mn occupation of 0.87(3)/0.13; (b) one complete γ-cluster; (c) the bcc unit cell; (d) the octahedral partial structure, representing the magnetic lattice with diluted Mn spins on the OH sites, and (e) the tetrahedral partial structure, representing the positional disorder in the partially populated CC and IT sites (both occupied by Zn).

The structure can be described in close analogy to the Ni_2_Zn_11_ parent γ-brass compound, in which Ni occupies solely the 8c(OT) position with full occupancy, whereas the remaining three sites 8c(IT), 12e(OH), and 24g(CO) are fully occupied by Zn. The pseudobinary structures C1 and C2 adopt the same structure type, described by the 26-atom γ-cluster. The refined site occupancies for the C1 composition reveal that the central (CC) site, which is empty in the γ-cluster of the Ni_2_Zn_11_, is partially occupied by Zn (SOF(CC) = 0.107(13)) with a simultaneous depletion of Zn at the IT sites (SOF(IT) = 0.861(7)). The OH site shows a Zn/Mn substitutional disorder. The OT and CO sites that are occupied by Ni and Zn, respectively, remain unaffected by the Mn insertion. The structure for the C1 composition is shown in Figure 2. The five successive shells of the distorted γ-cluster are presented in Figure 2a, with the OH sites showing mixed Zn/Mn occupation of 0.87/0.13, whereas one complete γ-cluster is shown in Figure 2b. The bcc unit cell is shown in Figure 2c, whereas the magnetic lattice with diluted Mn spins on the OH sites (see later) is shown in Figure 2d. The partial structure of the partially populated CC and IT sites (both occupied by Zn) is shown in Figure 2e. The C2 crystal shows a similar occupancy pattern and crystal structure, where the occupancy of the CC site slightly decreases (SOF(CC) = 0.082(11)), with a further increase of Zn occupation at the IT sites (SOF(IT) = 0.913(5)).

### 2.2 Spin-Glass Magnetism of the γ-Brass Type Mn_0.3_Ni_2_Zn_10.7_

Magnetic properties were studied via the magnetic experiments and the specific heat for the loading composition Mn_0.3_Ni_2_Zn_10.7_(C1). The structure contains two kinds of magnetic elements, namely, Mn and Ni, where Mn is antiferromagnetic (AFM) as a pure metal, whereas Ni is ferromagnetic (FM). In an electrically conducting medium, the interaction between the moments is the conduction-electron mediated Ruderman–Kittel–Kasuya–Yosida (RKKY) indirect exchange, which oscillates in space on the scale of many nanometers between positive and negative values, depending on the distance between the spins. Because of the random positioning of Mn on the octahedral partial structure (the Zn/Mn mixed-occupied OH site), both FM and AFM interactions are present, causing frustration of the magnetic subsystem. The disorder caused by a partial occupancy of the CC and IT sites adds to the frustration as well.

#### Temperature-Dependent Magnetization and Magnetic Susceptibility

**Figure 3:**
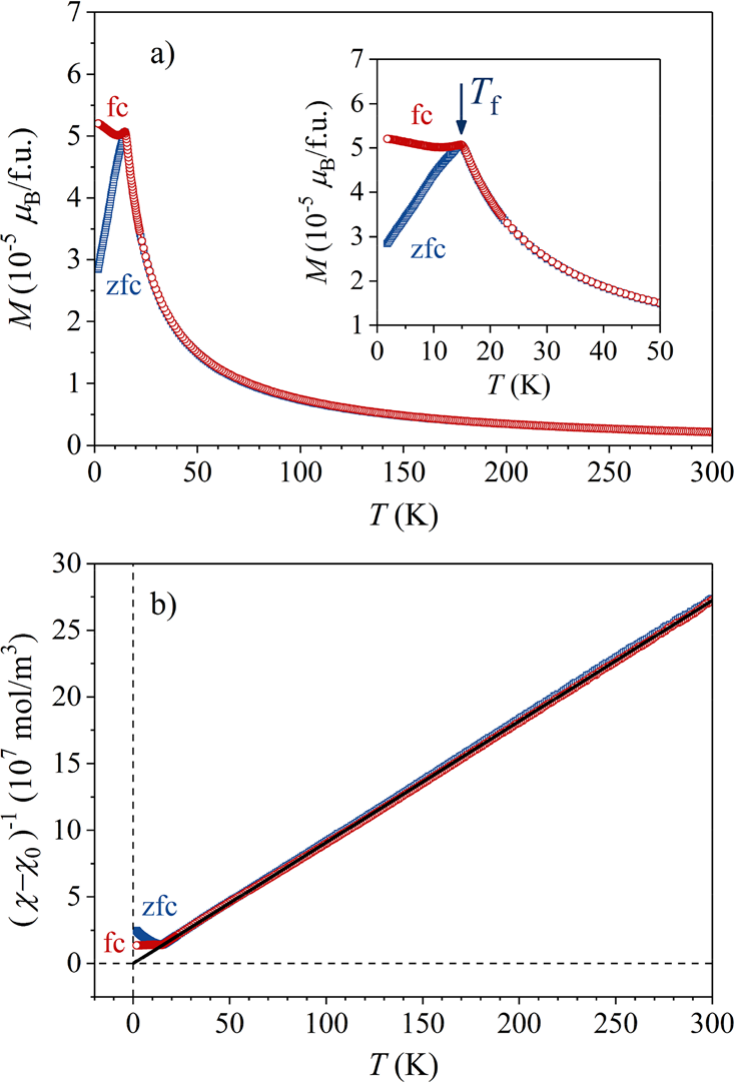
(a) Temperature-dependent zfc and fc dc magnetization in a magnetic field μ_0_*H* = 5 mT (fu denotes formula unit, that is, one Mn_0.033_Ni_0.148_Zn_0.819_ average “atom”). (inset) The transition region around the spin freezing temperature *T*_f_ on an expanded scale. (b) Magnetic susceptibility in a Curie–Weiss plot, where the solid line is the fit in the paramagnetic regime.

The temperature-dependent zero-field-cooled (zfc) and field-cooled (fc) direct-current (dc) magnetization in a low magnetic field of μ_0_*H* = 5 mT, measured in the temperature range of 300–1.8 K, is shown in Figure 3a. The magnetization is given in Bohr magnetons μ_B_ per one Mn_0.033_Ni_0.148_Zn_0.819_ average “atom” (i.e., per one formula unit (fu)). When cooled, a paramagnetic growth of the magnetization is observed down to 16 K, where *M*_zfc_ exhibits a cusp and decreases toward zero when further cooled, whereas *M*_fc_ stays approximately constant below that temperature. Such behavior is typical of a kinetic spin-freezing transition in spin glasses,^16^ where the temperature of the *M*_zfc_ cusp is associated with the spin-freezing temperature *T*_f_. Below *T*_f_, the ergodicity of the spin system is broken; that is, the spin fluctuation times become so long that the system cannot reach an equilibrium state on the accessible experimental frequency scale, which is the origin of the *M*_zfc_ – *M*_fc_ splitting.

The magnetic susceptibility χ = *M*/*H* in the paramagnetic phase (*T* > *T*_f_) was analyzed by the Curie–Weiss law, χ = χ_0_ + *C*_CW_/(*T* – θ). Here *C*_CW_ is the Curie–Weiss constant, θ is the Curie–Weiss temperature, and χ_0_ is the temperature-independent term of the susceptibility. χ_0_ is a sum of the negative (diamagnetic) Larmor core susceptibility, the positive Pauli paramagnetic spin susceptibility of the conduction electrons, and the negative Landau susceptibility due to the conduction-electron orbital circulation in the magnetic field, where all three contributions are of the same order of magnitude. The Larmor susceptibility was estimated from literature tables^17^ as *χ*_Larmor_ ≈ – 2 × 10^–10^ m^3^ mol^–1^. The fit yielded the value χ_0_ = −6.6 × 10^–10^ m^3^ mol^–1^, which is of the correct order of magnitude. The plot of the susceptibility (χ - χ_0_)^−1^ vs *T* and the Curie–Weiss fit (solid line) are shown in Figure 3b. Excellent fit was obtained in the entire paramagnetic regime above *T*_*f*_ ≈ 16 K. The mean effective Bohr magneton number *p̅*_eff_, obtained from^18^ has yielded the mean effective paramagnetic moment *μ̅*_eff_ = *p̅*_eff_*μ*_B_ = (0.84 ± 0.05)*μ*_B_. This value is strongly reduced with regard to the experimental paramagnetic free-ion values of localized ions Mn^3+^, Mn^2+^, Ni^3+^, and Ni^2+^ that amount to 4.9, 5.9, 4.8, and 3.2 μ_B_, respectively. On the basis of the Curie–Weiss analysis we are, however, unable to discriminate between a small number of magnetic ions with large magnetic moments and a large number of magnetic ions with small magnetic moments in the Mn_0.3_Ni_2_Zn_10.7_ structure. Likewise, we are unable to discriminate between the magnetic moments of Mn and Ni.

The Curie–Weiss temperature was determined from the fit as θ = 0 K (with an estimated error of ±1 K). This temperature is generally an indication of the dominant type of interspin interactions, being either AFM (θ < 0) or FM (θ > 0). The Mn_0.3_Ni_2_Zn_10.7_ 3d magnetic alloy is an exchange-dominated spin system, where the randomness of the Mn positioning on the magnetic sublattice introduces a distribution of the exchange coupling constants . The zero value of the Curie–Weiss temperature suggests that the distribution function  extends to both positive (, FM coupling) and negative (, AFM coupling)  values.^19^ It also indicates that the average exchange interaction is close to zero, , which is characteristic of spin glasses. This supports that the magnetic state below *T*_f_ ≈ 16 K is of a spin-glass type, where the AFM and FM interactions are both present in similar proportions.

**Figure 4:**
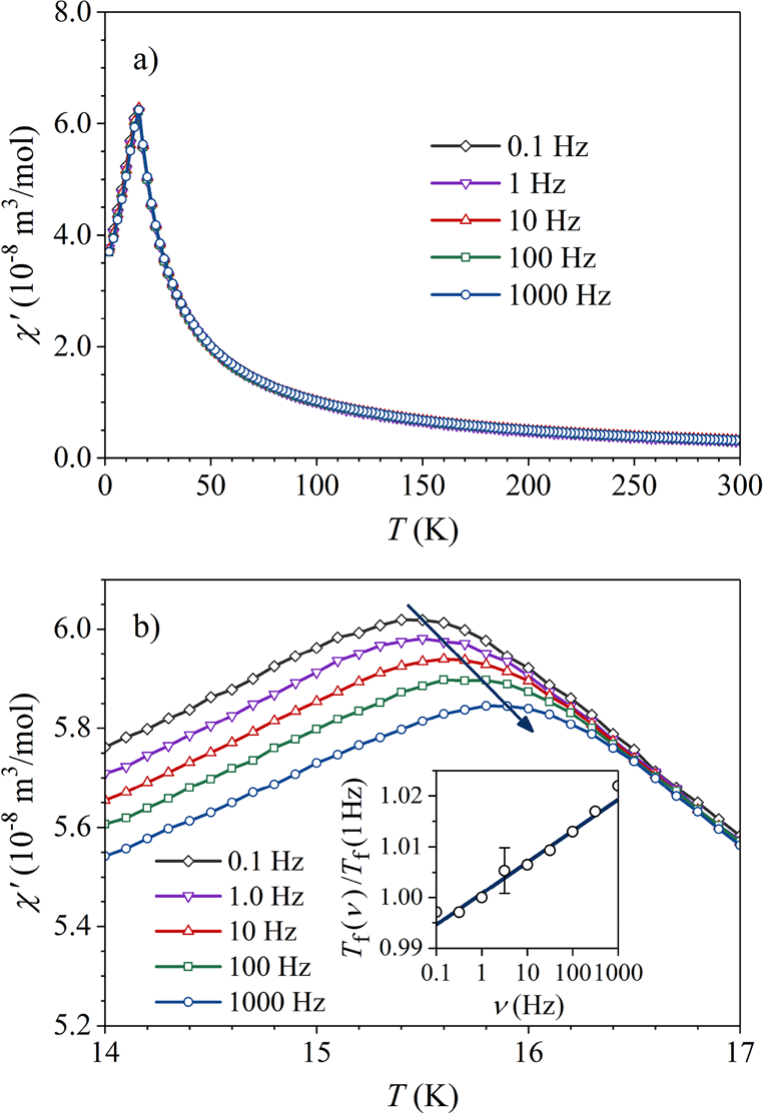
(a) Temperature- and frequency-dependent real ac susceptibility *χ*′. (b) The *χ*′ peak on an expanded scale (the arrow marks the shift of the peak with frequency). (inset) The *T*_f_ (ν)/*T*_f_ (1 Hz) vs ν relation.

The alternating-current (ac) magnetic susceptibility was measured between 300 and 1.8 K in a sinusoidal magnetic field of amplitude μ_0_*H*_0_ = 0.2 mT at logarithmically spaced frequencies between 0.1 and 1000 Hz. The real part of the susceptibility *χ*′ is presented in Figure 4a, where a peak at ∼16 K that shifts with frequency to higher temperatures is evident (the details are shown in Figure 4b). Such a frequency dependence indicates a spin-freezing transition from an ergodic to a nonergodic state. The frequency-dependent spin-freezing temperature *T*_f_ (ν) is conveniently defined as the temperature of the *χ*′ peak, and the corresponding *T*_f_ (ν)/*T*_f_ (1 Hz) relation is shown in the inset of Figure 4b. The fractional shift of Δ*T*_f_/*T*_f_ per log ν was determined as Γ = Δ*T*_f_/*T*_f_ Δ(log ν) = 6.1 × 10^–3^. This value falls into the range typical for spin glasses, where the values of Γ from 10^–2^ to 10^–3^ are common.^20^

#### The M(H) Magnetization

**Figure 5:**
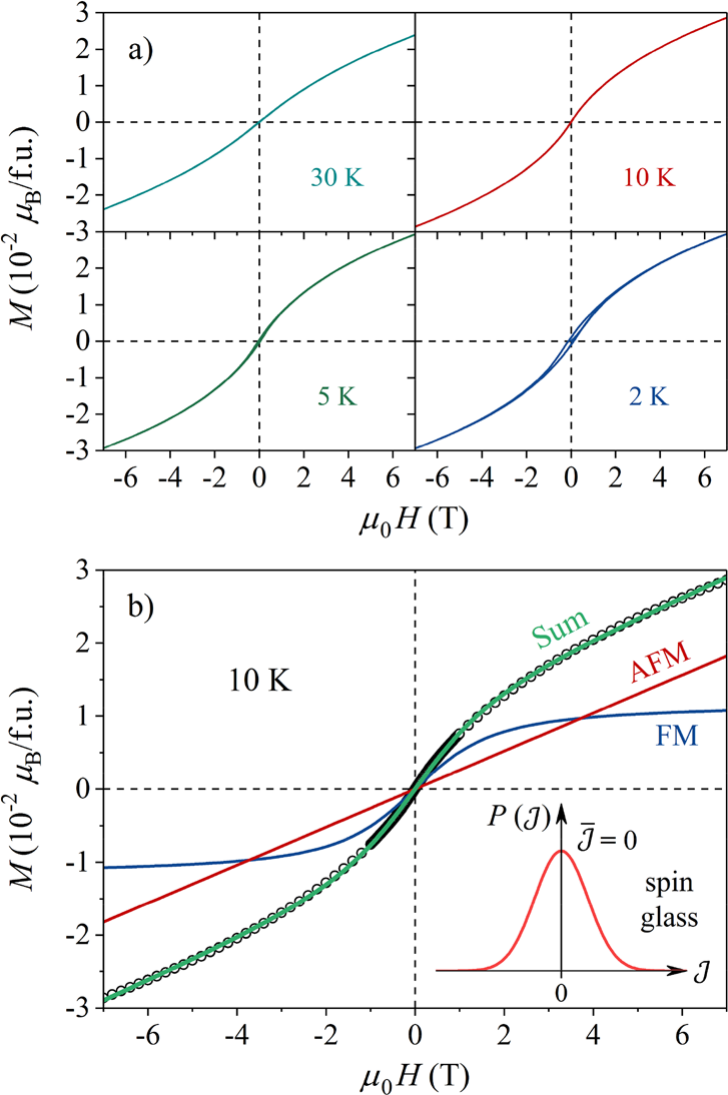
(a) *M*(*H*) curves in the temperature range of 30–2 K. (b) The 10 K curve with the theoretical fit (see text), showing also the FM and AFM contributions separately. (inset) Schematic view of the  distribution for a spin glass ().

The *M*(*H*) magnetization curves below 30 K are shown in Figure 5a. Hysteresis becomes noticeable below 10 K, where the coercive field at the lowest measured temperature of 2 K amounts to μ_0_*H*_c_ = 0.12 T. The close-up field of the hysteresis loops is ∼2 T. At large fields, the *M*(*H*) curves approach asymptotically an inclined line.

The 10 K *M* (*H*) curve, where the hysteresis is still absent, was theoretically reproduced by assuming a spin-glass-type distribution , shown schematically in the inset of Figure 5b. The curve was modeled by the function *M* = *M*_0_*L*(*x*) + *kμ*_0_*H*. Here the term *M*_0_*L*(*x*) corresponds to the  (FM) side of , where *L*(*x*) with *x* = *μμ*_0_*H*/(*k*_B_*T*) is the Langevin function, and μ is the effective moment of the FM clusters. The term *kμ*_0_*H* corresponds to the  (AFM) side of , with μ_0_*k* representing the AFM susceptibility. In the limit of large *H*, the total *M*(*H*) curve approaches asymptotically an inclined line of the slope μ_0_*k*.

The theoretical fit of the 10 K curve with the above model is presented in Figure 5b. The fit yielded the parameter values *M*_0_ = 1.2 × 10^–2^*μ*_B_/fu, μ = 22μ_B_, and *k* = 2.6 × 10^–3^*μ*_B_/(fuT). The small FM group moments of 22*μ*_B_ suggest FM spin domains of a couple of nanometers in size. The fit qualitatively confirms the spin-glass-type  distribution with vanishing average exchange interaction, . This is in accord with the zero Curie–Weiss temperature.

#### Specific Heat

Specific heat is suitable for the characterization of phase transitions (structural, magnetic, superconducting), where an ordering of some degree of freedom takes place at the phase transition temperature. The energy of the ordered state is lower than the energy of the high-temperature disordered state, so that the energy released upon ordering exits the system as a heat in the form of an exothermic peak. The low-temperature specific heat of a magnetic alloy can be written as *C* = *γT* + *αT*^3^ + *C*_m_, where *γT*, *αT*^3^, and *C*_m_ are the electronic, lattice, and magnetic contributions, respectively. For canonical spin glasses (denoting a noble metal host like Cu or Ag with diluted magnetic impurities Fe, Mn, etc.), *C*_m_ usually exhibits a broad peak upon spin freezing, with the peak maximum located at *T*_max_ ≈ 1.4*T*_f_.^21^ The exothermic peak in the region of the spin-freezing temperature indicates that the spins adjust locally to their neighbors in an energetically favorable configuration to reduce the exchange energy, although no long-range magnetic ordering takes place. For a purely kinetic spin freezing transition, however, the spins or small spin clusters gradually freeze into random directions during continuous cooling, so that no heat is released at *T*_f_, and the specific heat does not show any anomaly.

**Figure 6:**
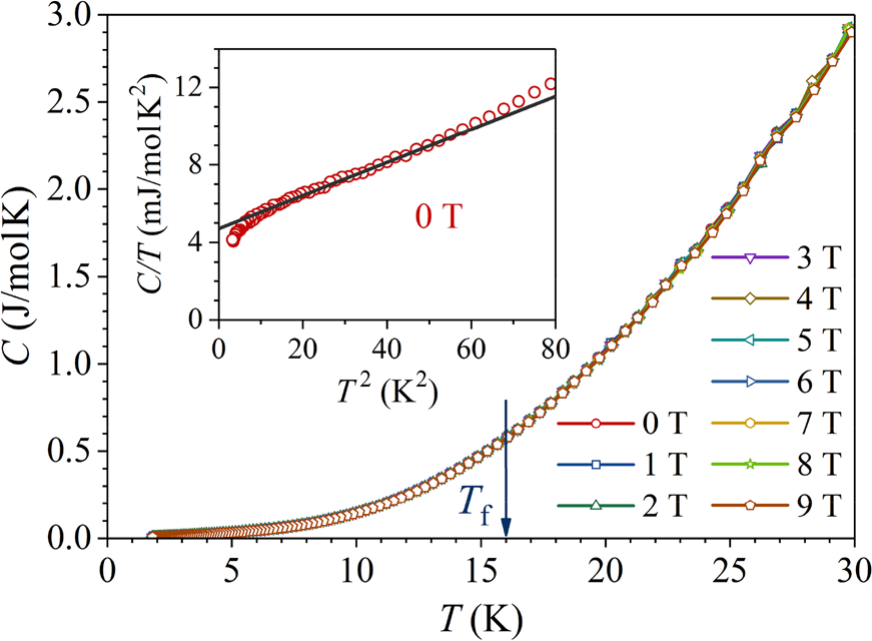
Specific heat *C*(*T*) below 30 K in the magnetic field range of 0–9 T. Vertical arrow denotes the spin-freezing temperature *T*_f_ = 16 K, determined from the magnetic susceptibility. (inset) The specific heat below 9 K in a *C*/*T* vs *T*^2^ plot.

The specific heat of the Mn_0.3_Ni_2_Zn_10.7_ γ-brass was determined in the temperature range of 1.8–300 K in magnetic fields between 0 and 9 T. The *C*(*T*) graphs below 30 K are shown in Figure 6, where it is evident that there is no anomaly at the spin-freezing temperature *T*_f_ ≈ 16 K, and the specific heat also does not show any field dependence. This is in favor of a kinetic spin-freezing transition from an ergodic to a nonergodic state. The specific heat below 10 K is presented in the inset of Figure 6 in a *C*/*T* versus *T*^2^ plot, wherefrom the electronic specific heat coefficient was determined as γ = 4.71 mJ mol^–1^ K^–2^. This value is almost the same as that reported for the Ni_2_Zn_11_ parent γ-brass (γ = 4.5 mJ mol^–1^ K^–2^).^22^ Since γ = (π^2^/3) *k*_B_^2^*g* (*ε*_F_) is directly proportional to the electronic DOS at the Fermi level *g* (*ε*_F_), this indicates that the DOS at *ε*_F_ of the Mn-doped Mn_0.3_Ni_2_Zn_10.7_ is not changed much with respect to the Ni_2_Zn_11_. The lattice specific heat coefficient α yielded the Debye temperature of *θ*_D_ = 283 K (for comparison, the *θ*_D_ values of the constituent metals are θ_D_^Zn^ = 329 K, θ_D_^Ni^ = 477 K, and θ_D_^Mn^ = 409 K).

#### Thermoremanent Magnetization

The broken-ergodicity state of a spin glass (as well as of other magnetically frustrated systems) can be further characterized by measuring the time decay of the thermoremanent magnetization (TRM), which is logarithmically slow below the spin-freezing temperature *T*_f_.^23−31^ This reflects the slow approach of the spin system toward a thermal equilibrium, which cannot be reached on the available frequency scale of a given experimental technique due to a broad distribution of spin relaxation times. The TRM time-decay measurement protocol involves (1) continuous cooling of the spin system in a magnetic field *H*_fc_ through *T*_f_ into the nonergodic state, (2) stopping the cooling at a certain measuring (or aging) temperature *T*_m_ < *T*_f_ for a waiting (aging) time *t*_w_, (3) cutting the field to zero after *t*_w_, and (4) recording the *M*_TRM_ magnetization decay as a function of time *t*. The TRM decay depends on *T*_m_, *t*_w_, and *H*_fc_.

**Figure 7:**
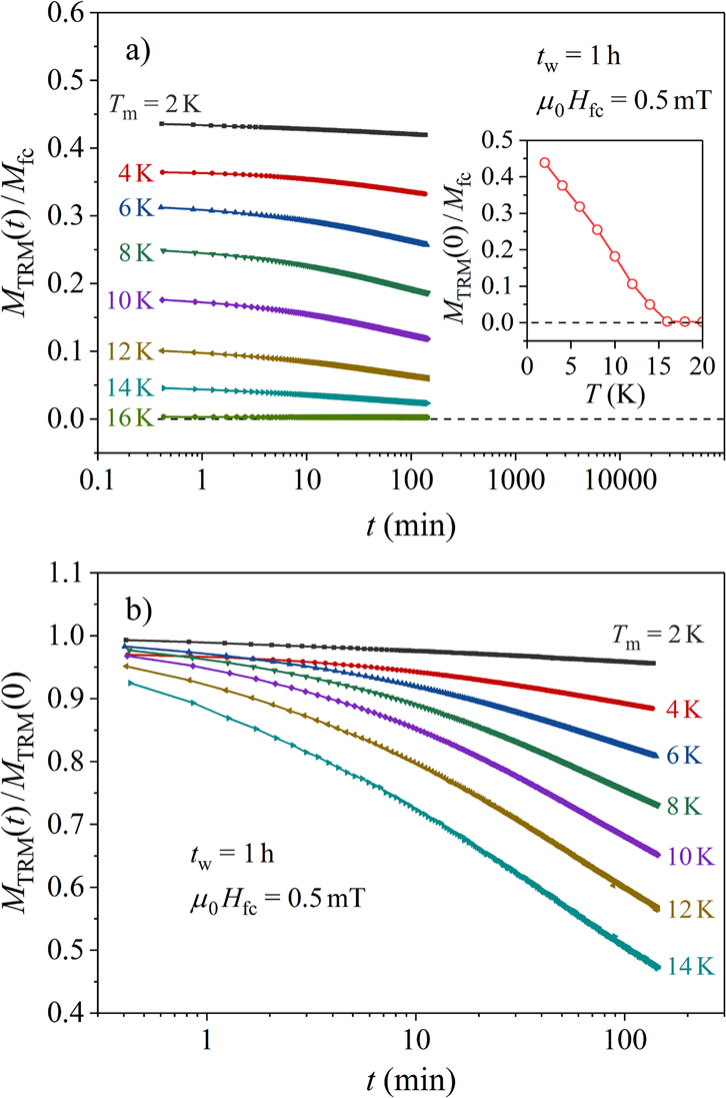
(a) Normalized TRM time-decay curves *M*_TRM_ (*T*_m_, *t*)/*M*_fc_ (*T*_m_), at different temperatures *T*_m_ below *T*_f_ ≈ 16 K. The normalized initial (*t* = 0) amplitude, *M*_TRM_ (*T*_m_, *t* = 0)/*M*_fc_ (*T*_m_) as a function of *T*_m_ is shown in the inset. (b) Each curve is shown normalized to its *t* = 0 amplitude, *M*_TRM_ (*T*_m_, *t*)/*M*_TRM_ (*T*_m_, *t* = 0).

The TRM versus *T*_m_ experiments, performed on the Mn_0.3_Ni_2_Zn_10.7_ γ-brass, are shown in Figure 7. A small field of μ_0_*H*_fc_ = 0.5 mT was applied, and the aging stops were performed at eight different temperatures *T*_m_ between 16 and 2 K in steps of Δ*T*_m_ = 2 K, where aging for *t*_w_ = 1 h was employed at each stop. After *H*_fc_ → 0, the TRM decays were recorded up to *t* ≈ 150 min. The normalized decays *M*_TRM_ (*T*_m_, *t*)/*M*_fc_ (*T*_m_), where *M*_fc_ (*T*_m_) is the fc magnetization just before the field cutoff, are shown in Figure 7a, where an increase in amplitude with the decreasing *T*_m_ is evident. The inset shows the normalized initial (*t* = 0) amplitude *M*_TRM_ (*T*_m_, *t* = 0)/*M*_fc_ (*T*_m_) as a function of *T*_m_. The TRM at *T*_m_ = *T*_f_ = 16 K is zero, because the spin system is ergodic at that temperature. The decays slow down strongly with decreasing *T*_m_, owing to an increased remanence of the spin system at lower temperatures. This is demonstrated in Figure 7b, where the TRM decays normalized to their *t* = 0 amplitudes, *M*_TRM_ (*T*_m_, *t*)/*M*_TRM_ (*T*_m_, *t* = 0), are shown.

**Figure 8:**
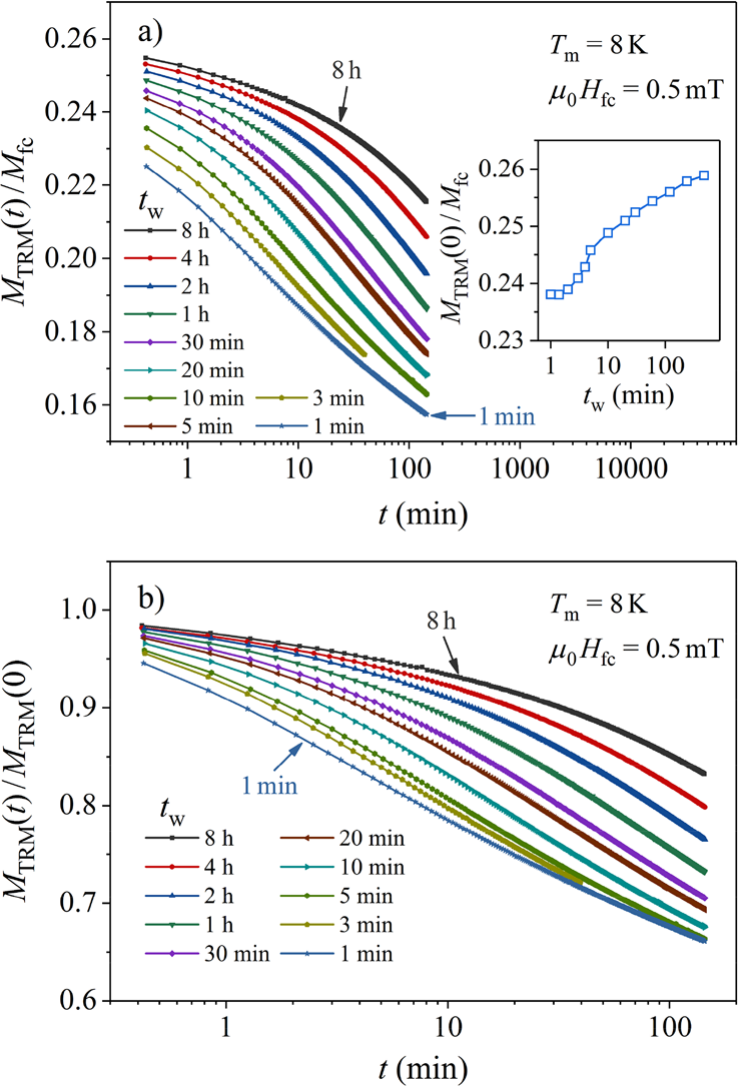
(a) Normalized TRM time-decay curves *M*_TRM_(*t*_w_*,t*)/*M*_fc_(*t*_w_) as a function of the aging time *t*_w_ at *T*_m_ = 8 K. The normalized initial (*t* = 0) amplitude, *M*_TRM_(*t*_w_*,t* = 0)/*M*_fc_ (*t*_w_), as a function of *t*_w_ is shown in the inset. (b) Each curve is shown normalized to its *t* = 0 amplitude, *M*_TRM_(*t*_w_*,t*)/*M*_TRM_(*t*_w_*,t* = 0).

The TRM time decays versus the aging time *t*_w_ at *T*_m_ = 8 K, obtained after field cooling in μ_0_*H*_fc_ = 0.5 mT, are presented in Figure 8. Eight different aging times between 1 min and 8 h, roughly logarithmically spaced, were employed. The normalized decay curves *M*_TRM_(*t*_w_*,t*)/*M*_fc_(*t*_w_) are shown in Figure 8a. The TRM increases for longer *t*_w_, which is additionally demonstrated in the inset of Figure 8a, where the normalized initial (*t* = 0) TRM amplitude, *M*_TRM_(*t*_w_*,t* = 0)/*M*_fc_(*t*_w_), is shown as a function of *t*_w_. The decay shapes also change with *t*_w_, where a slowing-down for longer *t*_w_ values is evident. This is demonstrated in Figure 8b, where the TRM decays normalized to their *t* = 0 amplitudes, *M*_TRM_(*t*_w_*,t*)/*M*_TRM_(*t*_w_*,t* = 0), are shown.

**Figure 9:**
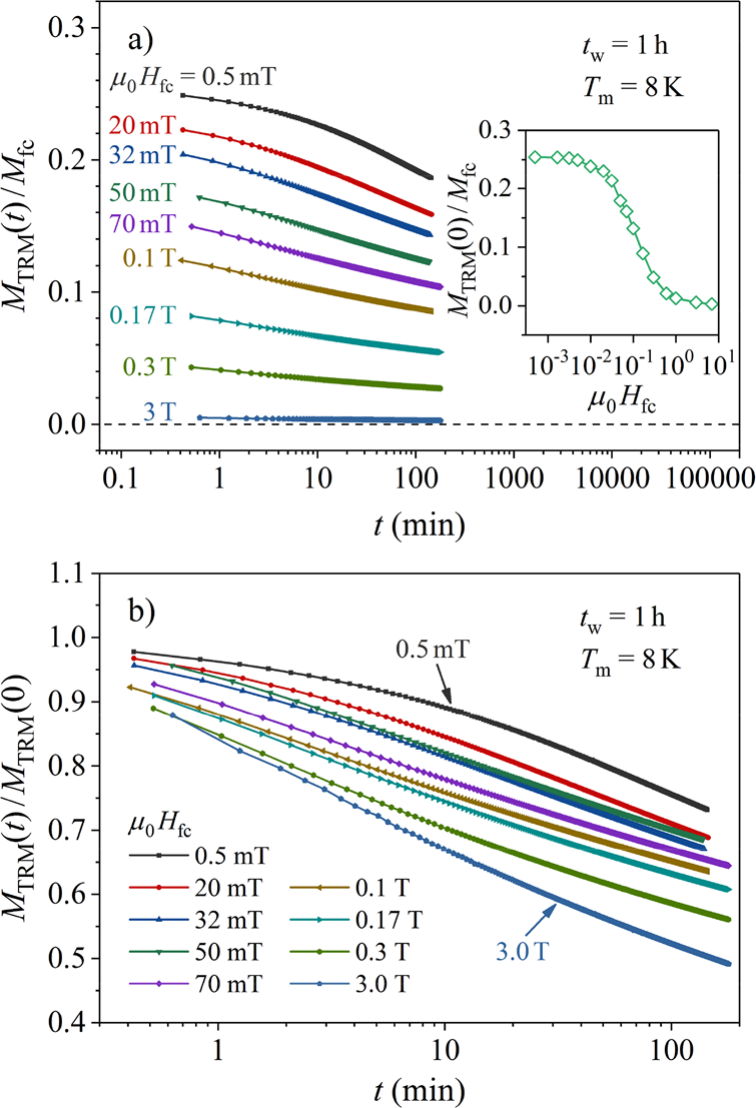
(a) Normalized TRM time-decay curves *M*_TRM_ (*H*_fc_*,t*)/*M*_fc_ (*H*_fc_) vs the field *H*_fc_, measured at *T*_m_ = 8 K for the aging time *t*_w_ = 1 h. The normalized initial (*t* = 0) amplitude *M*_TRM_ (*H*_fc_*,t* = 0)/*M*_fc_ (*H*_fc_) as a function of *H*_fc_ is shown in the inset (note that the field scale is logarithmic). (b) Each curve is shown normalized to its *t* = 0 amplitude, *M*_TRM_ (*H*_fc_*,t*)/*M*_TRM_ (*H*_fc_,t = 0).

The TRM time decays versus the field *H*_fc_, measured at *T*_m_ = 8 K for the aging time *t*_w_ = 1 h, are shown in Figure 9a. Fifteen field values between μ_0_*H*_fc_ = 0.5 mT and 7 T, roughly logarithmically spaced, were used. The normalized TRM time decays, *M*_TRM_ (*H*_fc_,*t*)/*M*_fc_ (*H*_fc_), demonstrate a strong decrease of the TRM with increasing *H*_fc_. The inset in Figure 9a shows the normalized initial (*t* = 0) TRM amplitude *M*_TRM_ (*H*_fc_,*t* = 0 )/*M*_fc_ (*H*_fc_), as a function of *H*_fc_. A large decrease by 2 orders of magnitude within the investigated field range is evident. The change of the shape of the decay curves with *H*_fc_ is presented in Figure 9b, where each curve is shown normalized to its *t* = 0 amplitude, *M*_TRM_ (*H*_fc_*,t*)/*M*_TRM_ (*H*_fc_,*t* = 0). The decays show a tendency to slow down for smaller *H*_fc_ values.

#### Memory Effect

The memory effect (ME)^27,30,32−35^ is another manifestation of the out-of-equilibrium, ultraslow dynamics of a nonergodic spin system, where the spin state formed upon isothermal aging can be retrieved after a reverse temperature cycle. The ME measurement protocol involves (1) continuous cooling of the spin system in a zero magnetic field through *T*_f_ into the nonergodic phase, (2) stopping at a certain temperature *T*_m1_ < *T*_f_, and letting the system age isothermally for a macroscopic time *t*_w_, ranging from a few minutes to many hours, (3) resuming the zero-field cooling after *t*_w_, by performing eventually one or more additional consecutive aging stops at lower temperatures *T*_mi_, (4) applying a tiny magnetic field of the order μ_0_*H* = 0.1 mT at the lowest temperature, and (5) recording the zfc magnetization *M*_zfc_ in a continuous heating run. The spin system remembers the isothermal aging periods at all stop temperatures *T*_mi_, by showing a diminution (a dip) in the *M*_zfc_ at each *T*_mi_, with respect to the no-aging case (*t*_w_ = 0). In addition to the aging temperatures, the spin system remembers also the duration *t*_w_ of each aging stop. By heating into the ergodic phase, all the memorized information is erased, and the spin system is the same as before aging, a phenomenon known as rejuvenation.

**Figure 10:**
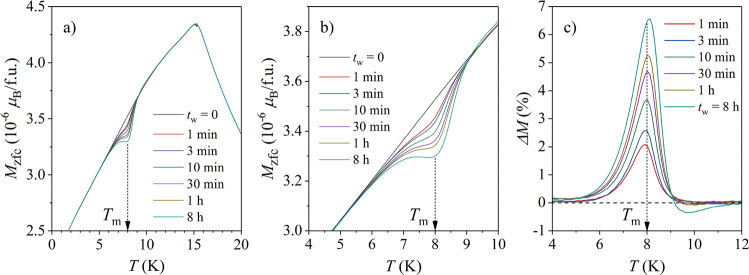
(a) *M*_zfc_ curves for different aging times *t*_w_ between 1 min and 8 h at *T*_m_ = 8 K. (b) *M*_zfc_s in the region of *T*_m_ on an expanded scale. (c) The normalized difference Δ*M* = [*M*_zfc_ (*t*_w_ = 0) – *M*_zfc_(*t*_w_)]/*M*_zfc_ (*t*_w_ = 0).

The ME on the Mn_0.3_Ni_2_Zn_10.7_ γ-brass was performed by stopping at *T*_m__1_ = 8 K and employing a series of aging times between *t*_w_ = 1 min and 8 h, approximately logarithmically spaced (each *t*_w_ was used in a separate experiment). A no-stop reference run (*t*_w_ = 0) was also performed. At 2 K (the lowest temperature of the cooling run), a field μ_0_*H*_zfc_ = 0.5 mT was applied. The *M*_zfc_ values for all *t*_w_ values, measured when heated, are shown in Figure 10a, where a dip at *T*_m__1_ in the aged curves is observed. The *M*_zfc_s in the vicinity of *T*_m__1_ are shown on the expanded scale in Figure 10b, where it is seen in more detail how the dip increases with the increasing *t*_w_. The normalized difference between the reference (*t*_w_ = 0) curve and the aged curves, Δ*M* = [*M*_zfc_ (*t*_w_ = 0) – *M*_zfc_(*t*_w_)]/*M*_zfc_ (*t*_w_ = 0), is shown in Figure 10c. Δ*M* is peaked at the aging temperature *T*_m__1_ and increases in amplitude with *t*_w_.

**Figure 11:**
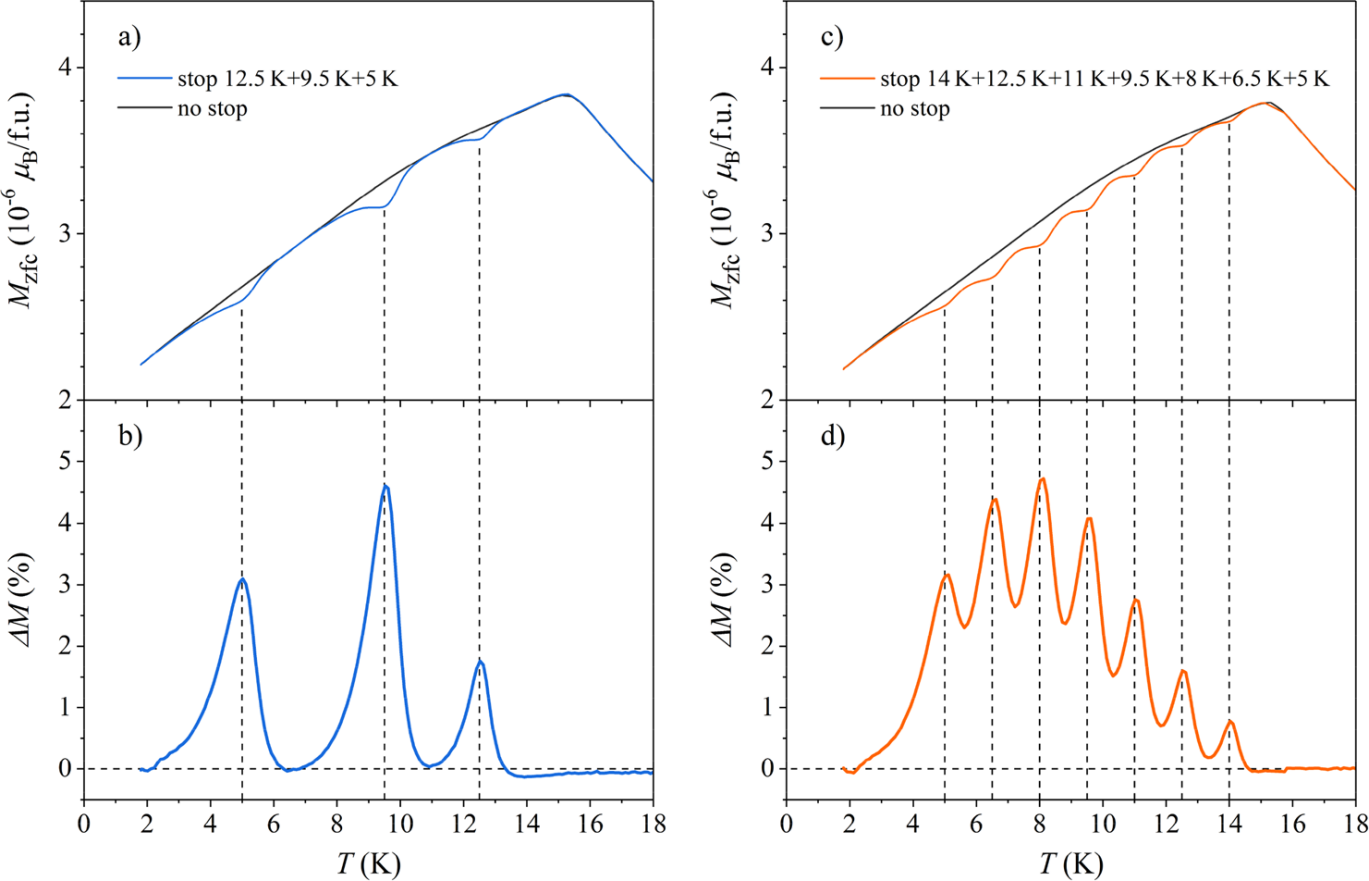
(a) *M*_zfc_ curve for three consecutive stops at *T*_m__1_ = 12.5 K, *T*_m__2_ = 9.5 K, and *T*_m__3_ = 5 K for *t*_w_ = 1 h at each stop. The no-stop reference run is also presented. (b) Normalized difference Δ*M*, where three peaks at the *T*_mi_ values are evident. (c) *M*_zfc_ for seven consecutive stops at *T*_mi_ (*i* = 1–7) between 14 and 5 K separated by Δ*T*_mi_ = 1.5 K and (d) the corresponding Δ*M*s.

The ME involving multiple consecutive isothermal aging stops for *t*_w_ = 1 h within the same zfc cooling run was observed by performing three stops at *T*_m__1_ = 12.5 K, *T*_m__2_ = 9.5 K, and *T*_m__3_ = 5 K. The no-stop reference run was also recorded. The corresponding *M*_zfc_s are presented in Figure 11a, showing a dip at each *T*_mi_. The normalized difference Δ*M* with three well-resolved peaks at *T*_mi_ is shown in Figure 11b. The *M*_zfc_s for seven consecutive stops for *t*_w_ = 1 h each at the temperatures *T*_mi_ (*i* = 1–7) between 14 and 5 K in steps of Δ*T*_mi_ = 1.5 K are shown in Figure 11c, whereas the corresponding Δ*M*s are shown in Figure 11d. All seven consecutive aging stops have been memorized by the nonergodic spin system.

## 3 Discussion

Randomness, as one of the reasons responsible for the spin-glass behavior of the Mn_0.3_Ni_2_Zn_10.7_ γ-brass, is emerging from the random distribution of diluted Mn moments at the OH sites, because Ni occupies the OT sites with the full occupancy of 1, and there is no randomness in the Ni sublattice. Another source of randomness is the structural disorder caused by a partial occupancy of the CC and IT sites, though both sites are populated by nonmagnetic Zn. Here it is not clear whether both Mn and Ni ions possess sizable localized magnetic moments and participate in the spin-glass magnetism or only Mn is magnetic. A partial answer to this question can be derived from the specific heat study performed on the Ni_2_Zn_11_ parent γ-brass,^22^ which has revealed that no long-range magnetic ordering of Ni moments takes place down to 0.35 K. The reported *C/T* = 4.5 mJ mol^–1^ K^–2^ value in the *T* → 0 limit can be attributed entirely to the electronic specific heat (the electronic specific heat coefficients of the Ni and Zn metals are *γ*_Ni_ = 7.04 mJ mol^–1^ K^–2^ and *γ*_Zn_ = 0.64 mJ mol^–1^ K^–2^), so that there is no magnetic specific heat, suggesting that Ni ions do not possess sizable magnetic moments. Doping with a small amount of Mn is not expected to change significantly the magnetic state of Ni in the Mn_0.3_Ni_2_Zn_10.7_ pseudobinary γ-brass, so it is reasonable to assume that the magnetism of the Mn-doped compound originates predominantly from the Mn ions. The diluted Mn moments are positioned randomly on the octahedral partial structure. The long-range oscillating RKKY interaction then frustrates the Mn spins, which are subjected to competing AFM/FM interactions, and a spin-glass state is consequently formed.

The magnetic state of the Mn_0.3_Ni_2_Zn_10.7_ γ-brass exhibits broken-ergodicity phenomena below the spin-freezing temperature that are typical for spin glasses and other magnetically frustrated spin systems. The memory effect has already found an important technological application as a thermal memory cell for the thermal storage of digital information, in the absence of an electric, magnetic, or electromagnetic field.^36^ The observed broken-ergodicity phenomena are not restricted to spin glasses but are present also in other nonergodic magnetically and electrically frustrated systems. A theoretical understanding of these out-of-equilibrium phenomena is still incomplete, because the experimentally measured physical quantities are frequency-dependent, depending on the frequency scale of the employed experimental observation technique. A comprehensive literature on the subject exists,^16,25−36^ and for details, the reader is invited to consult those articles. Here we emphasize that the TRM and the ME in the Mn_0.3_Ni_2_Zn_10.7_ γ-brass exhibit the same type of the *T*_m_, *t*_w_, and *H*_fc_ dependence as reported before for other spin systems belonging to the broad class of spin glasses, either diluted or concentrated, electrically conducting or insulating, site-disordered or site-ordered with geometric frustration. Such systems include canonical spin glasses,^16,27−30,33,34^ geometrically frustrated quasicrystals,^31,37^ and complex metallic alloys,^32^ high-entropy alloys,^35,38,39^ and magnetic nanoparticles.^40,41^ The Mn_0.3_Ni_2_Zn_10.7_ pseudobinary γ-brass can be classified as a spin glass, representing the first alloy from the family of γ-brasses belonging to this class.

## 4 Conclusions

The Mn_*x*_Ni_2_Zn_11–*x*_ (*x* = 0.1–0.5) pseudobinary γ-brass type phases were synthesized by a high-temperature synthesis. The crystal structures for two nonstoichiometric loading compositions *x* = 0.3 and 0.5 were determined by XRD and solved in the cubic space group *I*43*m*. Like the binary Ni_2_Zn_11_ parent γ-brass, the structures are described by the 26-atom γ-cluster, distributed on a bcc lattice. The 8c(OT) and 24g(CO) sites of the γ-cluster are occupied solely by Ni and Zn, respectively, whereas the 12e(OH) site is Zn/Mn mixed populated. The 2a(CC) and 8c(IT) sites show a positional disorder, both being partially occupied by Zn. According to the space group type and the combination of chemical elements, the investigated Mn_*x*_Ni_2_Zn_11–*x*_ compounds belong to the *I*-cell γ-brasses from the group II.

It is worth mentioning that, due to the similar X-ray scattering factors of the constituent elements (Mn, Ni, and Zn), a site occupancy and substitution pattern could be accurately addressed by a combination of X-ray and neutron diffraction analyses. The neutron powder diffraction analysis of the γ-brass type pseudobinary phases will be the subject of our future investigations.

Magnetic properties were studied for the low-Mn loading composition of Mn_0.3_Ni_2_Zn_10.7_. On the basis of the measurement of the temperature-dependent zfc and fc magnetization, the ac susceptibility, the *M*(*H*) hysteresis curves, the thermoremanent magnetization, and the memory effect, we found that the spin system exhibits typical broken-ergodicity phenomena of a magnetically frustrated spin system below the spin-freezing temperature *T*_f_ ≈ 16 K. Specific heat measurements support a kinetic spin-freezing transition. The above results allow a classification of the Mn_0.3_Ni_2_Zn_10.7_ pseudobinary γ-brass as a spin glass.

## 5 Experimental Section

Samples of several loading compositions Mn_*x*_Ni_2_Zn_11–*x*_(*x* = 0.1–0.5) were prepared from high-purity elements Mn (99.98%), Ni (99.99%), and Zn (99.999%) from Alfa Aesar. The amounts of elements were weighted according to the loading compositions and kept in one-end-closed silica tubes. The other ends of the ampules were sealed under high vacuum (pressure < 10^–5^ mbar). Sealed ampules were placed into a programmable furnace, which was heated to 600 °C at a rate of 2 °C/min, and then kept at that temperature for 24 h. After that, the samples were heated to 900 °C at a rate of 0.2 °C/min and annealed at that temperature for 6 h, followed by a cool-down to 450 °C at a rate of 0.1 °C/min. Next, they were annealed at that temperature for 107 h. A rapid cooling was then performed to 100 °C at a rate of 1.2 °C/min and kept at that temperature for 54 h. Finally, the furnace was turned off and left to cool naturally to room temperature.

The phase purity was verified for all samples by PXRD. A small fraction of each ingot was crushed and ground into a fine powder using an agate mortar and pestle. The PXRD data were collected at ambient temperature using a Rigaku MiniFlex 600 diffractometer (Bragg–Brentano geometry) with a 600 W X-ray tube (Cu Kα_1_ radiation, λ = 1.540 56 Å) and D/teX Ultra silicon strip detector. The data were collected for the 2θ range from 10° to 80° with a step size of 0.02°. The JANA2006 program^42^ was used to refine the PXRD data.

The structural model was elaborated from SCXRD data and analysis. Single crystals were selected from crushed ingots of the loading compositions Mn_0.3_Ni_2.0_Zn_10.7_ and Mn_0.5_Ni_2.0_Zn_10.5_, for which the PXRD patterns are shown in Figure 1. The crystals were glued to the tip of a glass fiber and mounted on the goniometer head. Each data set was collected at room temperature with a Bruker Photon II diffractometer equipped with graphite-monochromatic Mo Kα radiation (λ = 0.710 73 Å). The data reduction was performed by Apex III software. The structure solution and refinement were made by using the JANA2006 package program.^42^

An EDS analysis of the chemical composition was performed on Tungsten Environmental Scanning Electron Microscope ESEM Quanta 650 equipped with an Oxford Instruments Aztec Live Ultim Max SDD 40 mm2 EDS detection system.

Magnetic measurements were conducted on a Quantum Design MPMS3 magnetometer, equipped with a 7 T magnet and operating at temperatures between 1.8 and 400 K. The thermoremanent magnetization in low magnetic fields and the memory effect experiments were conducted using a copper AC/ULF coil of the MPMS3 magnetometer to ensure an accurate and repeatable magnetic field. Prior to the low-field experiments, the Ultra-Low Field option was used to compensate for the residual magnetic field of the superconducting magnet. A needle-shaped sample of dimensions 5 × 1 × 1 mm^3^ was cut from the ingot and contained several differently oriented crystal grains. The long axis was parallel to the magnetic field, to minimize the demagnetization effect. The specific heat was measured on a Quantum Design Physical Property Measurement System (PPMS 9T), equipped with a 9 T magnet and operating in the temperature range between 1.9 and 400 K.

## Accession Codes

CCDC 2078889–2078890 contain the supplementary crystallographic data for this paper. These data can be obtained free of charge via www.ccdc.cam.ac.uk/data_request/cif, or by emailing data_request@ccdc.cam.ac.uk, or by contacting The Cambridge Crystallographic Data Centre, 12 Union Road, Cambridge CB2 1EZ, UK; fax: +44 1223 336033.
